# Effects of daily almond consumption on glycaemia in adults with elevated risk for diabetes: a randomised controlled trial

**DOI:** 10.1017/S0007114524001053

**Published:** 2024-11-28

**Authors:** Li-Chu Huang, Gregory C. Henderson, Richard D. Mattes

**Affiliations:** Department of Nutrition Science, Purdue University, West Lafayette, IN, USA

**Keywords:** Almond, HbA1c, Diabetes/Prediabetes, Obesity, Dietary Intervention

## Abstract

The purpose of this study was to examine the potential for sustained almond consumption to reduce HbA1c concentrations among individuals with elevated values. A 16-week randomised, parallel-arm, controlled trial was conducted. Eighty-one adults with elevated HbA1c concentrations (> 5·7 %) were randomly assigned to incorporate 2 oz of raw almonds (A: *n* 39) or energy-matched snacks (C: *n* 42) into their daily diets. Body weight, body composition, plasma lipids, HbA1c, plasma vitamin E, glycaemia (by meal tolerance test and continuous glucose monitoring), dietary intake and hedonic responses to test foods were measured at stipulated time points. Participants consuming almonds ingested 253 kcal/d more than participants in the control group (*P* = 0·02), but this did not result in a significant difference in body weight. No statistically significant differences were observed in HbA1c concentrations, blood chemistries, body composition or glycaemia over time or between groups. However, Healthy Eating Index scores improved within the almond group as compared with the control group (*P* < 0·001). Additionally, the hedonic rating of almonds within the almond group did not decline as markedly as the control group’s reduced liking of the pretzel snack. Alpha-tocopherol increased significantly, and gamma tocopherol tended to decrease in the almond group, indicating compliance with the dietary intervention. Overall, daily ingestion of 2 oz of raw almonds in a self-selected diet for 16 weeks did not alter short-term or longer-term glycaemia or HbA1c concentrations in adults with elevated HbA1c concentrations, but they were well-tolerated hedonically and improved diet quality without promoting weight gain.

The escalating prevalence of type 2 diabetes mellitus (T2D) poses a significant health concern, impacting individuals and straining healthcare systems nationally^(1,2)^ and globally^(3)^. An estimated 11·6 % of Americans (∼38 million) have diabetes and 38% (∼98 million) have prediabetes^(4)^. More than 80% of individuals with prediabetes remain undiagnosed, allowing continuation of lifestyle choices that may promote the progression to T2D and postponing efforts to implement lifestyle choices that may reduce risk^(5)^. Dietary interventions that seek to improve glycaemic management are a cornerstone of approaches to mitigate the growth of this pandemic^(6)^. Almonds, a nutrient-dense tree nut, have a low glycaemic index and have a protein, soluble fibre, and monounsaturated fat profile that may hold beneficial effects on glycaemia^(7)^. Previous acute trials have demonstrated almonds lower post-prandial glycaemia^(8–12)^ and evoke a second meal effect^(13)^, especially when consumed at breakfast or as mid-morning or mid-afternoon snacks^(13,14)^. However, the efficacy of sustained almond consumption for reducing glycated hemoglobin (HbA1c) concentrations, a critical marker of long-term glycaemic control, remains uncertain.

The inconclusive evidence regarding the impact of prolonged almond consumption on HbA1c may stem from discrepancies in research methodologies. Two trials with durations of fewer than 8 weeks unsurprisingly did not detect alterations in HbA1c concentrations^(15,16)^, as its measurement reflects the red blood cell turn-over rate of 2–3 months^(17)^. Other trials lasting ≥ 12 weeks have shown significant reductions, albeit with limited sample sizes^(10)^ or of only a marginal reduction of HbA1c (< 1 %)^(18)^. Thus, intervention duration is a critical methodological consideration. Another methodological concern is the baseline HbA1c status of study participants. Some studies examining the effects of almond consumption for more than 24 weeks have not detected significant differences in HbA1c concentrations, possibly because participants had normal pre-intervention values^(19–21)^. Expectations that a mild dietary intervention will alter normal values may be unrealistic, whereas those with higher concentrations, arguably the target population, may be more likely to manifest reductions through almond consumption. To date, two studies employing robust methodological designs to assess the potential impact of sustained almond consumption on HbA1c concentrations among patients with prediabetes or T2D have yielded inconclusive findings^(22,23)^. These studies provided almonds to substitute for 20 % of total energy intake for 16 or 24 weeks among individuals with elevated HbA1c concentrations. One study reported a reduction in HbA1c concentrations (based on individuals with T2D)^(22)^, while the other did not (based on individuals with prediabetes)^(23)^. Whether the effect of almonds on individuals with T2D is more pronounced remains to be elucidated. Importantly, another methodological concern with the existing literature is the inclusion of weak compliance measures leaving open questions about whether the low or absent effects are accurate or reflect poor adherence to the dietary prescription thereby weakening the test of the hypothesis. The study that demonstrated an effect relied solely on self-reported questionnaires and phone calls^(22)^, while the other study that did not observe an effect used food diaries with additional objective compliance measures (i.e. increased plasma *α*-tocopherol), although data are provided for only the early phase of the study^(23)^. Additionally, the quantity of almonds administered is critical. An influence on HbA1c concentration has been noted at a portion as low as < 1 oz/d^(10)^, but the magnitude of effect appears to be dose dependent where a dose of ≥ 2 oz elicits a more pronounced response^(24)^. This quantity is greater than the mean nut intake among the American population^(25)^.

Some interventions have implemented dietary protocols and restrictions to accommodate the high energy content of the prescribed almonds. The study with the greatest reduction in HbA1c (12 %, percentage reduction from baseline) among individuals with T2D was designed to offset 2 ounces of almonds with a 150 g reduction of staple foods, while adhering to meals meeting diabetes dietary guidelines over a period of 12 weeks^(26)^. Another study conducted over 24 weeks also reported a small, but significant decrease in HbA1c levels (–4 %) where participants were instructed to displace 20 % of their total energy intake from discretionary fat and carbohydrates with almonds, alongside receiving repeated exercise counseling^(22)^. However, access to such specialised dietary support may be limited due to factors such as cost and availability. Hence, assessing the efficacy of almonds in improving glycaemic control under less controlled conditions, where individuals may not have access to personalised dietary guidance, is warranted.

The primary aim of this study was to clarify the potential for chronic (16 weeks of daily intake) almond consumption, at a moderate daily quantity (i.e. 2 oz/d) without additional dietary guidance on the customary diet, to lower fasting and post-prandial glycaemia and HbA1c concentrations in individuals with elevated HbA1c concentrations. Secondary aims were to assess whether almond consumption could improve diet quality, body weight and body composition. The study was conducted in free-living individuals, and dietary compliance was measured objectively.

## Methods

### Study population

Participants were recruited between January 2022 and February 2023. Eligibility criteria included men and women with HbA1c ≥ 5·7 %, BMI ≥ 20 kg/m^2^, 18–70 years of age, no or stable medication use for > 3 months, no tree nut or peanut allergy, good dentition, ≥ 4 eating events per day and consumption of ≥ 1 low nutrient density snack/day. Exclusion criteria included smoking and pregnancy. Participants were randomised to the almond group or control group using a random number sequence (generated at randomizer.org). This study was conducted according to the guidelines laid down in the Declaration of Helsinki, and all procedures involving human subjects/patients were approved by the Institutional Review Board at Purdue University (IRB-2021-1008). Written informed consent was obtained from all participants. This study is registered at clinicaltrials.gov with identification number: NCT05176197 (https://classic.clinicaltrials.gov/ct2/show/NCT05176197).

### General protocol

This was a 16-week randomised, controlled, parallel arm clinical trial. Prospective participants completed an online pre-screening questionnaire. Those meeting preliminary eligibility criteria were scheduled for an in-person screening session where blood samples were collected for determination of HbA1c concentrations and measurements were made of height and weight.

Participants assigned to the almond group were provided two 1 oz (28 g) packets of unsalted raw almonds to consume twice a day: one pack with their habitual breakfast and one as a replacement of their mid-morning or mid-afternoon snack. The total amount of almonds consumed per day was ∼56 g, which provided 328 kcals. For those who were assigned to the control group, two packs of energy-matched, unsalted pretzels (Snyder’s of Hanover Mini Unsalted Pretzels) were provided with the identical consumption guidelines as the almond group. The nutrient composition of the almonds and pretzels in a 1-serving portion are shown in Table 1. Both groups were additionally instructed not to consume any other nuts or nut products and to remain on their customary diet, physical activity pattern and medications (if applicable) throughout the trial. Assessments performed during the trial included: body weight, fasting blood draws, meal tolerance test, body composition, hedonic ratings, dietary recalls and continuous glucose monitoring (CGM).

**Table 1.** Nutrient comparison of the intervention foods. Values are presented per serving. The total quantity of prescribed intervention foods is two servings daily, with one serving consumed at breakfast and the other serving replacing the mid-morning or mid-afternoon snack

Name	Serving size	kcal	Carbohydrate (g)	Fat (g)	Protein (g)	Fiber (g)	Sugar (g)	Sodium (mg)
Almonds	28 g (24 nuts)	164	6·1	14·2	6	3·5	1·2	0
Pretzels	41·7 g (18 minis)	164	34·3	0·7	4·5	1·5	1·5	0

Values are presented per serving. Daily intake was two servings.

### Anthropometrics

Height was measured at baseline with a stadiometer (Seca, Chino, CA; Model 213 1821009). Body weight was measured at baseline and weeks 4, 8, 12 and 16 (Tanita, Arlington Heights, IL; Model TBF-410) with participants wearing undergarments or a hospital gown. Dual X-ray absorptiometry (Lunar DPX-IQ 240 densitometer version Encore GE 15, GE Healthcare) was used for body composition analysis. Dual X-ray absorptiometry measurements were conducted at baseline and week 16. CoreScan™ software was applied to quantify visceral adipose tissue (VAT) content in the android region (area between the ribs and the pelvis) using geometric calculations and attenuation measurements. Subcutaneous adipose tissue (SAT) was calculated by total android fat minus VAT. VAT ratio was calculated by VAT divided by total android fat. SAT ratio was calculated by SAT divided by total android fat.

### Biochemical assays

Fasting blood draws (HbA1c, glucose, insulin, C-peptide, total cholesterol, TAG, HDL-cholesterol and vitamin E) were performed at baseline and weeks 8 (HbA1c and vitamin E only) and 16; a meal tolerance test (meal-stimulated glucose, insulin and C-peptide) was conducted at baseline and week 16. Fasting blood samples were obtained after an overnight fast (≥ 10 h) via venipuncture. All samples were aliquoted and stored at −80° Celsius until analysed in batch. For the meal tolerance test, participants were instructed to consume an 8-ounce nutrition shake containing 19 g of carbohydrate, 2 g of fat and 16 g of protein (Ensure high protein nutrition shake – milk chocolate, Abbott Laboratories, Chicago, IL) within 10 min. Additional blood samples were obtained 10, 20, 30, 60, 120 and 180 min after shake consumption.

HbA1c was analysed in whole blood; glucose, insulin, C-peptide and lipids were assayed in serum and vitamin E was measured in plasma. HbA1c, glucose, TAG, total cholesterol and HDL-cholesterol were determined on a Roche COBAS Integra 400 Plus analyser (Roche Diagnostics Corporation). The lower detection limit and repeatability of the assays are 0·18 mmol/l and CV of 0·8 % for HbA1c; 4·32 mg/dl and CV of 0·6 % for glucose; 8·85 mg/dl and CV of 1·6 % for TAG; 3·87 mg/dl and CV of 0·7 % for total cholesterol and 3 mg/dl and CV of 0·8 % for HDL-cholesterol. Insulin and C-peptide were measured on a Roche COBAS E411 analyser (Roche Diagnostics Corporation, Indianapolis, IN). The lower detection limit and repeatability of the assays were 0·2 μU/ml and CV of 1·3 % for insulin; sensitivity of 0·01 ng/ml and CV of 1·1 % for C-peptide. Incremental AUC was calculated using the trapezoidal method, with any values below baseline omitted. LDL-cholesterol concentrations were determined using the Friedewald formula: LDL-cholesterol (mg/dL) = total cholesterol – HDL-cholesterol – (TAG/5)^(27)^. Insulin resistance and the homeostatic model assessment for insulin resistance (HOMA-IR) were computed using the formula, HOMA-IR = [fasting glucose (mg/dl) × fasting insulin (μU/ml)]/405^(28)^. Homeostasis model assessment of *β*-cell function (HOMA-%B), which serves as an index for insulin secretory function, was derived using the formula HOMA-%B = 360 × fasting insulin (μU/ml)/(Fasting glucose (mg/dl) – 63)^(28)^. The primary outcome measure, HbA1c concentration, was assessed repeatedly, with screening and baseline values averaged, and blood samples collected twice at week 16, with the averages calculated to mitigate variability and enhance statistical power.

### Continuous glucose monitoring

Continuous glucose monitoring (CGM) (FreeStyle Libre Pro System) was used to monitor day-long blood glucose concentrations at baseline and weeks 8 and 16. The CGM sensor was placed on the back upper arm, and readings were made every 15 min for 24 h. Standard foods and beverages were not provided during the CGM measurement to allow for the observation of glucose fluctuations under free-living conditions. CGM data were analysed as 24-hour AUC, maximum and minimum glucose concentrations as well as the mean glucose concentration and measures of glycaemic variability over the 24-hour period, the daytime period (06.00–00.00) and the nighttime period (00.00–06.00). The 24-hour AUC was calculated using the trapezoidal method.

### Dietary recalls and quality

Two non-consecutive days of dietary recalls were conducted with a registered dietitian at baseline and weeks 8 and 16. These included one weekday and one weekend day. The data were analysed with the web-based ‘Minnesota Nutrition Data System for Research (NDS-R version 2022)’. The Goldberg formula^(29)^ was used to estimate plausible reporters with the determination approach adapted from prior work^(30)^. Diet quality was assessed by a Healthy Eating Index-2015 (HEI-2015) score for each participant using the Legacy SAS code provided by the Nutrition Coordinating Center^(31)^. The mean values within each week were computed.

### Hedonic ratings

Hedonic ratings of intervention foods were acquired using a visual analog scale administered through the online Qualtrics XM survey platform (Seattle, WA) at baseline and weeks 4, 8, 12 and 16. Participants were asked to taste one almond and one pretzel and rate their liking of each by marking a lab iPad (iPad mini-2, ME276LL/A). They were instructed to rinse their oral cavity with filtered water for a duration of 5 s and then expectorate and sample one stimulus. Immediately following this, participants rated their liking for the sampled item using a 0–100-point visual analog scale on the screen. The scale featured two descriptive anchors, ‘Dislike extremely’ at 0 and ‘Like extremely’ at 100, with the numerical values concealed from the participants to maintain blinding throughout the evaluation process. They then waited for 10 s before undertaking the same process with the alternate stimulus. The order of sample testing was randomised.

### Compliance

Both self-reported and objective compliance measures were used to ascertain participants’ adherence to the study protocol. Self-reported compliance was calculated by the total number of days participants reported consuming both intervention foods divided by the total number of intervention days. Objective compliance was assessed using established protocols for measurement of plasma vitamin E as detailed in prior studies^(32,33)^. Plasma vitamin E was assayed using HPLC.

### Statistical analysis

Group differences in baseline characteristics were compared by independent *t*-tests for continuous variables and χ^2^ tests for categorical outcomes. Intention-to-treat analyses were conducted on all participants who provided baseline data (intention-to-treat, *n* 81). A linear mixed model was used to determine the effect of time, treatment and treatment × time interaction for each variable using SPSS (version 29) with no imputation for missing values. Time and treatment as well as the interaction were treated as fixed effects, and participants were treated as random effects repeated over time using a repeated covariance matrix. In each analysis, when main or interaction effects were significant, pairwise comparisons with Bonferroni correction were applied. When data were not normally distributed, extreme outliers (> 4 standard deviations) were removed from the analyses. The sample size was calculated using power analyses, which indicated a sample of thirty-three participants per group would be sufficient to detect a 10 % decrease in HbA1c with 90 % power at the 5 % level of probability. For all analyses, statistical significance was determined at the *α* level of 0·05. Data are reported as mean and standard error of the mean (sem) unless otherwise stated.

## Results

### Study population

**Fig. 1.**
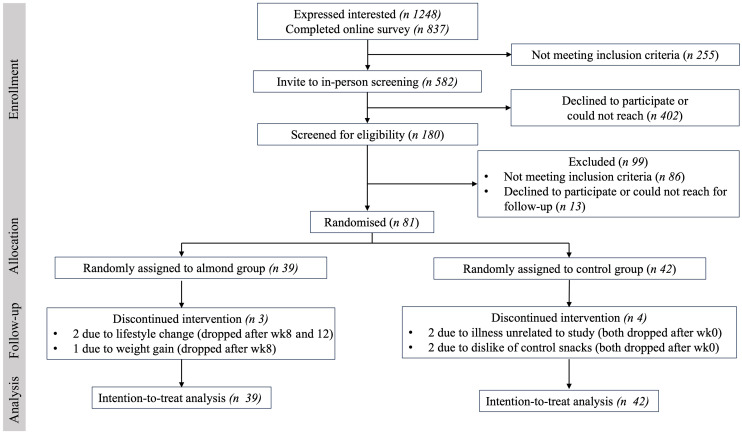
Participant flow chart. Final intention-to-treat analyses was conducted on all participants who provided baseline data.

One hundred and eighty individuals were screened for eligibility and eighty-one enrolled and were randomised to a study group (Fig. 1). Three participants withdrew from the almond group (attrition rate = 7·7 %), and four participants withdrew from the control group (attrition rate = 9·5 %). Baseline characteristics in both groups including dropout rates, sex, race, age, BMI, body weight, HbA1c or fasting glucose concentration are presented in Table 2. Participants were primarily Caucasian females with higher BMI, HbA1c and fasting glucose concentrations than general healthy populations.

**Table 2.** Baseline characteristics. Data are presented as count and the percentage for categorical variables or mean (sem) for continuous variables (Numbers and percentages; mean values with their standard error of the mean)

	Almond		Control	
	Counts		Counts	
*n*	39		42	
Dropped out	3		4	

	*n*	%	*n*	%
Sex				
Male (%)	12	30·8	11	26·2
Female (%)	27	69·2	31	73·8
Race				
Caucasian (%)	30	76·9	36	87·8
Asian (%)	6	15·4	3	7·3
African American (%)	1	2·6	2	4·9
Hispanic (%)	2	5·1	1	2·4

	Mean	sem	Mean	sem
Age (years)	50·1	2·2	48·8	1·8
Body weight (kg)	97·3	4·1	103·5	3·4
BMI (kg/m^2^)	34·2	1·2	36·2	1·2
HbA1c (%)	6·7	0·3	6·6	0·3
Fasting glucose (mg/dl)	122·7	9·0	125·2	9·0

### Blood biochemistry

None of the biochemical indices assessed differed between groups at baseline (Table 3). There were no significant main effects or time-by-treatment interactions for HbA1c or fasting glucose, fasting insulin, fasting C-peptide, meal-stimulated insulin, meal-stimulated C-peptide, total cholesterol, TAG, LDL-cholesterol, HOMA-IR or HOMA-%B (Table 3). There was a significant main effect of treatment on meal-stimulated glucose incremental AUC (*P* < 0·001), where the almond group had a higher value than the control group. There was no significant main effect of time or a treatment-by-time interaction (Both *P* > 0·05). There was a main effect of time on HDL-cholesterol (*P* = 0·01), where the value was increased significantly at week 16 compared with week 0, with no significant main effect of treatment or a treatment-by-time interaction (both *P* > 0·05). The usage of diabetes-related medications was considered as a covariate but did not reach statistical significance and did not influence the study outcomes, prompting their exclusion from the final statistical model. A *post hoc* analysis was conducted with independent *t*-tests, including only participants with baseline HbA1c levels ≥ 6·5 %, which is a commonly recommended cut point for diagnosing diabetes (A: *n* 15; C: *n* 12). Results showed a trend toward lower HbA1c levels (Appendix Table 1, *P* = 0·08) and a significantly lower fasting glucose (Appendix Table 1, *P* = 0·05) in the almond group compared with the control group. Another sub-analysis with independent *t*-tests revealed significant reductions in total cholesterol (data not shown, *P* = 0·04) and LDL-cholesterol concentrations (data not shown, *P* = 0·003) in the almond group compared with the control group.

**Table 3.** Blood biochemical indices. All data are presented as mean (sem) with each unit listed (Mean values with their standard error of the mean)

	Almond (*n 39*)						Control (*n 42*)								
	0		8		16		0		8		16		*P*-value^*^		
Week	Mean	sem	Mean	sem	Mean	sem	Mean	sem	Mean	sem	Mean	sem	*Tx*	*Time*	*Tx × Time*
HbA1c (%)	6·6	0·2	6·5	0·2	6·7	0·2	6·6	0·3	6·7	0·3	6·6	0·2	0·85	0·95	0·24

	0			16			0			16			*P*-value^*^		
Week	Mean	sem		Mean	sem		Mean	sem		Mean	sem		*Tx*	*Time*	*Tx × Time*
Fasting glucose (mg/dl)	115·6	5·6		120·7	6·8		125·2	9·0		125·5	8·5		0·31	0·17	0·83
Fasting insulin (μU/ml)	20·3	2·7		23·0	4·2		18·1	1·4		22·2	2·6		0·71	0·07	0·93
Fasting C-peptide (ng/ml)	3·0	0·2		3·0	0·3		3·1	0·2		3·0	0·2		0·96	0·46	0·65
Total CHOL (mg/dl)	163·5	6·3		161·1	6·1		173·6	5·4		177·3	4·8		0·15	0·37	0·74
TAG (mg/dl)	135·5	8·7		146·6	12·2		144·2	8·1		143·3	8·7		0·78	0·29	0·32
LDL-cholesterol (mg/dl)	93·0	5·4		86·4	5·1		103·2	4·9		106·6	4·3		0·06	0·80	0·29
HDL-cholesterol (mg/dl)	43·5	2·8		45·4	2·8		41·6	1·6		42·1	1·6		0·45	0·01	0·50
MTT glucose iAUC (min × mg/dl)	2979	349		3149	490		1613	161		1460	170		<0·001	0·94	0·52
MTT insulin iAUC (min × μU/ml)	4967	492		4519	586		4584	508		4773	548		0·80	0·39	0·47
MTT C-peptide iAUC (min × ng/ml)	327	28		280	29		278	25		283	37		0·56	0·07	0·49
HOMA-IR	6·1	0·8		6·1	1·2		5·8	0·7		6·6	0·8		0·91	0·37	0·75
HOMA-%B	180·7	27·2		166·3	23·5		165·7	16·8		159·5	14·5		0·59	0·14	0·97

*Tx*, treatment; CHOL, cholesterol; MTT, meal tolerance test; iAUC, incremental area under the curve.

Outliers (> 4 sd) removed from HbA1c (*n* 2), fasting glucose (*n* 1), insulin (*n* 1), HOMA-IR (*n* 3), glucose iAUC (*n* 1) and C-peptide iAUC (*n* 1) analyses. One data point removed from HOMA-%B (not applicable for the calculation). Missing data from HbA1c (*n* 1).

* Significant level of fixed effects by linear mixed model.

### Anthropometrics

There were no main effects or treatment-by-time interactions observed for body weight, BMI (Appendix Table 2), total fat mass, total lean mass, android mass, android fat mass, android lean mass, VAT, VAT ratio, SAT or SAT ratio (Appendix Table 3). A *post hoc* analysis was conducted with independent *t*-tests, including only participants with baseline HbA1c concentrations ≥6·5 %, (A: *n* 15; C: *n* 12). Results showed a trend towards lower body weight (data not shown, *P* = 0·09) in the almond group compared with the control group.

### Continuous glucose monitoring

There were no main effects or treatment-by-time interactions observed for mean daily glucose, the 24-hour glucose AUC, all-day CV, daytime CV, nighttime CV, mean daytime glucose, mean nighttime glucose, maximum or minimum 24-hour glucose concentrations (Appendix Table 4).

### Dietary intake

There were significant treatment-by-time interactions for energy, carbohydrate, fat, protein, added sugar, total fibre, vitamin E, as well as SFA, MUFA and PUFA intake (Appendix Table 5). Participants in the almond group consumed significantly more energy at week 16 compared with weeks 0 and 8 (Appendix Fig. 1, wk0 *v*. wk16:360·3 kilocalories (kcal), 95 % CI 156·1, 564·4, *P* < 0·001; wk8 *v*. wk16:320·0 kcal, 95 % CI 115·8, 524·2, *P* < 0·001) as well as 253 kcal/d more compared with the control group (286·8, 95 % CI 43·7, 530·0, *P* = 0·02). Energy intake did not differ significantly within the control group over time. Sixty-seven percent of the reported energy intake values fell within the Goldberg cut-offs (2SD).

Participants in the almond group consumed a lower percentage of total energy from carbohydrate (wk16 *v*. wk0: −5·0, 95 % CI −8·4, −1·5, *P* = 0·002), more from fat (wk16 *v*. wk0:5·2, 95 % CI 2·2, 8·2, *P* < 0·001) and no change for protein (*P* > 0·1). Participants in the control group consumed a higher percentage of daily energy from carbohydrate (wk16 *v*. wk0:6·0, 95 % CI 2·7, 9·4, *P* < 0·001), less from fat (wk16 *v*. wk0: −6·1, 95 % CI −9·0, −3·2, *P* < 0·001) and no change from protein (*P* > 0·1). The absolute intake of protein was significantly increased in the almond group (wk16 *v*. wk0:19·3 grams (g), 95 % CI 8·6, 30·0, *P* < 0·001) but not in the control group (*P* > 0·1). Total fibre (wk16 *v*. wk0:3·0 g, 95 % CI 0·2, 5·8, *P* = 0·03) and vitamin E were significantly increased in the almond group (wk16 *v*. wk0:13·0 milligrams, 95 % CI 11·3, 14·7, *P* < 0·001), while added sugar was decreased in the control group (wk16 *v*. wk0: −13·8 g, 95 % CI −26·7, −0·9, *P* = 0·03).

Participants in the almond group consumed a higher percentage of total energy from MUFA over time (wk16 *v*. wk0:5·3, 95 % CI 3·9, 6·7, *P* < 0·001), while participants in the control group consumed a lower percentage of energy from MUFA (wk16 *v*. wk0: −2·0, 95 % CI −3·4, −0·6, *P* = 0·002) and PUFA (wk16 *v*. wk0: −1·3, 95 % CI −2·6, −0·02, *P* = 0·05) during the intervention. The absolute intake of SFA in the almond group was significantly higher than the control group (A *v*. C: 9·9 g, 95 % CI 4·4, 15·4, *P* < 0·001).

### Diet quality

**Fig. 2.**
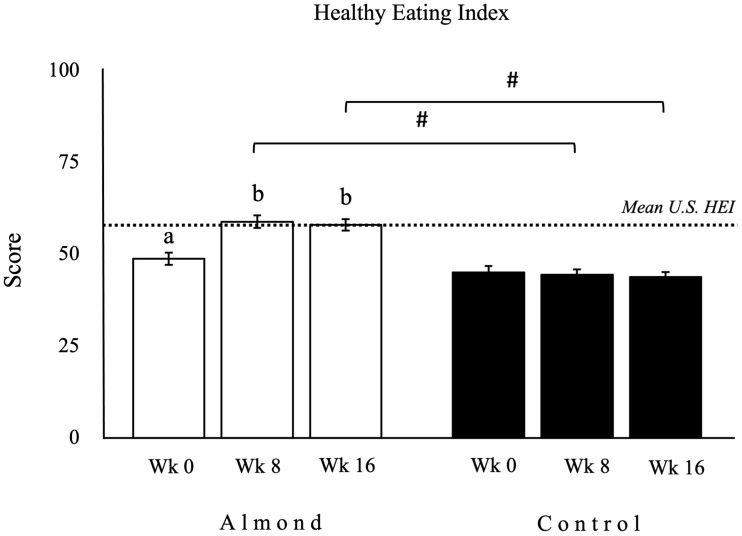
Healthy eating index (HEI) was significantly higher at weeks 8 and 16 compared with week 0 within the almond group (*n* 39) and as compared with the control group (*n* 42). Data for HEI are presented as mean (sem), with units expressed as 0–100 score. □ as the almond group; ▪ as the control group. ^ab^Different letters indicate a significant difference compared with week 0 within a group. ^#^Symbol indicates a significant difference between groups at the same time point. ···· Dotted line indicates the mean HEI score in the USA population aged 2 and older (58/100)^(34)^. Significance is defined as *P* < 0·05.

There was a significant treatment-by-time interaction for HEI score (Appendix Table 5). Participants in the almond group had a significantly higher total HEI score at weeks 8 and 16 compared with week 0 (Fig. 2, wk8 *v*. wk0:10·1, 95 % CI 6·0, 14·1, *P* < 0·001; wk16 *v*. wk0:8·2, 95 % CI 4·1, 12·4, *P* < 0·001), as well as compared with the control group at the corresponding time points (Fig. 2, A *v*. C wk8:15·1, 95 % CI 10·8, 19·4, *P* < 0·001; A *v*. C wk16:14·2, 95 % CI 9·8, 18·6, *P* < 0·001). There were no differences in total HEI scores over time among participants in the control group (Appendix Table 5, *P* > 0·1).

### Hedonic ratings

**Fig. 3.**
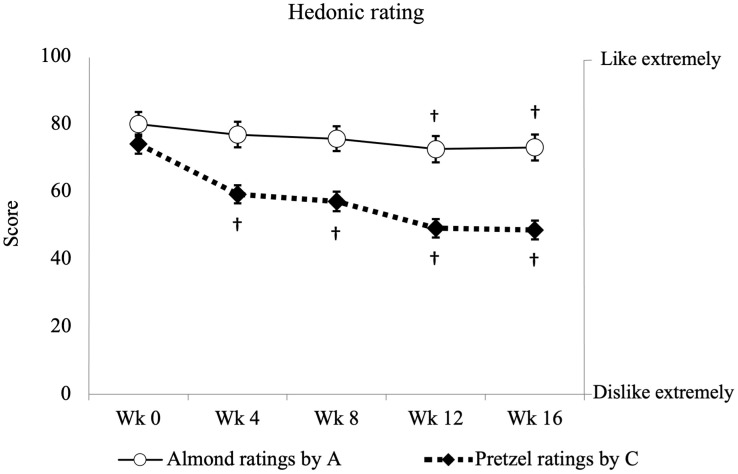
Hedonic ratings for the almond dropped slightly in the almond group (*n* 39), whereas the ratings for the pretzel declined significantly in the control group (*n* 42). Data for hedonic ratings are presented as mean (sem), with units expressed as 0–100 score. ○ as almond ratings by the almond group; ◆ as pretzel ratings by the control group. Y-axis on the left presents the score blinded to the participants; Y-axis on the right presents the descriptive anchors showed to the participant. ^†^ Symbol indicates a significant difference from the baseline within the same group. Significance is defined as *P* < 0·05.

There were significant treatment-by-time interactions for the hedonic ratings for almonds (*P* = 0·002) and for pretzels (*P* < 0·001). The hedonic ratings for almonds significantly declined at weeks 12 and 16 compared with week 0 in the almond group (Fig. 3, wk16 *v*. wk0: –8·9, 95 % CI: –15·9, –1·9, *P* = 0·004), while no change of the almond ratings was observed in the control group (*P* > 0·05). The between-group difference of the almond ratings was lower in the almond group compared with the control group at weeks 12 and 16 (data not shown, wk 16 A *v*. C: –12·9, 95 % CI –22·0, –4·0, *P* = 0·01). Conversely, the hedonic ratings for pretzels significantly declined at weeks 4, 8, 12 and 16 compared with week 0 in the control group (Fig. 3, wk16 *v*. wk0: –25·4, 95 % CI –35·0, –15·9, *P* < 0·001), with no observed change on the pretzel ratings in the almond group (*P* > 0·05). The between-group difference of the pretzel ratings was higher in the control group compared with the almond group at week 0 (data not shown, wk 0 C *v*. A: 12·3, 95 % CI 0·2, 24·3, *P* = 0·05), but lower at week 12 and 16 (Data not shown, wk 16 C *v*. A: –14·5, 95 % CI –26·8, –2·2, *P* = 0·02). The total reduction in almond ratings by the almond group was 9 %, whereas for pretzel ratings by the control group the reduction was 34 %. In contrast, the change in pretzel ratings in the almond group and the almond ratings in the control group were unchanged.

### Compliance

There was a significant treatment-by-time interaction for plasma *α*-tocopherol (Appendix Table 6). Plasma *α*-tocopherol significantly increased at weeks 8 and 16 compared with week 0 in the almond group (Appendix Table 6, wk8 *v*. wk0:3·1 micromolar (μM), 95 % CI 1·4, 4·7, *P* < 0·001; wk16 *v*. wk0:2·1 μM, 95 % CI 0·5, 3·8, *P* = 0·007). There was a trend for a treatment-by-time interaction for *γ*-tocopherol (Appendix Table 6, *P* = 0·09), with plasma *γ*-tocopherol tending to decrease in the almond group. No significant differences were observed for *α*-tocopherol or *γ*-tocopherol concentrations in the control group over time (*P* > 0·05). The mean self-reported compliance rates were 95 % for the almond group and 93 % for the control group.

## Discussion

Whether sustained almond consumption can lower HbA1c concentrations, particularly in individuals with elevated baseline concentrations, remains unclear. In this study, incorporating 2 ounces of almonds daily for 16 weeks did not result in a significant change of HbA1c concentration or other measures of glycaemic control nor in cardiometabolic risk factors. One possible explanation for this null finding could have been that study participants did not comply with the almond intervention. However, we do not believe this to be the case. First, because almonds are a rich source of *α*-tocopherol, while other *α*-tocopherol rich food sources are generally uncommon in the USA^(35)^, plasma concentrations have been shown to increase after almond supplementation^(36)^ and have been used as a marker for assessing adherence to an almond intervention^(15,19,23,37)^. In this trial, plasma *α*-tocopherol concentrations increased significantly only after almond consumption suggesting that study participants were compliant with the protocol. Additionally, the trend for decreasing plasma *γ*-tocopherol at week 16 in the almond group further supports compliance, as the circulating *γ*-tocopherol concentrations are inversely associated with total tocopherol intakes and circulating *α*-tocopherol concentrations^(38,39)^ due to their binding affinity for transfer proteins^(40)^. In contrast, no significant changes of *α*- or *γ*-tocopherol were observed in the control group. Additionally, the alterations in reported dietary macronutrient composition between the almond group (carbohydrate decreased by 5·0 %; fat increased by 5·0 %) and the control group (carbohydrate increased by 6·0 %; fat decreased by 6·0 %) were consistent with the inherent nutrient profiles of the intervention foods. This pattern of change aligns with findings from prior research^(19,23,26,37,41)^. Self-report compliance ratings were also high. While these latter indices of dietary compliance are not quantitative, they indicate the intervention was implemented and an alternate explanation of the findings is required. The two remaining possibilities are that conditions for identifying a contribution of almond intake on HbA1c reductions were not optimised or that almonds actually exert little or no effect on HbA1c concentrations. A critical consideration of the present and previous trial findings suggests the former may be the case.

First, previous studies reporting beneficial effects of almond consumption on HbA1c concentrations were often, but not uniformly^(23,37)^, accompanied by supplementary dietary consultation or dietary/lifestyle prescriptions. Shorter (i.e. 12 weeks^(26,42,43)^) and longer-term (i.e. 24 weeks^(22)^) trials that noted a significant reduction of HbA1c complemented the almond intervention with more comprehensive dietary and lifestyle guidance. In contrast, research reporting no discernible almond effect on HbA1c concentrations was often, though not exclusively^(10,18)^, those conducted with participants receiving either minimal or no dietary guidance^(41,44)^. In the present trial, no dietary guidance other than the expected consumption of the almonds or pretzels was provided. This may have limited our ability to detect an almond effect on HbA1c concentrations. Hence, the impact of almonds on HbA1c concentrations may be more apparent against a backdrop of a more healthful, energy-balanced overall diet.

A second potentially important consideration for assessing an effect of almond intake on HbA1c is a concomitant loss of body weight. A body weight reduction was observed in most almond intervention studies reporting reductions in HbA1c concentrations^(10,22,26,42)^. Weight loss itself is commonly associated with improved insulin sensitivity and long-term glycaemic control^(45–48)^. Systematic reviews and meta-analyses report that achieving a weight loss exceeding 5 % or more from lifestyle interventions aids the reduction of HbA1c^(49)^ with the magnitude of improvement increasing in proportion to the extent of body weight reduction^(50)^. Importantly, improvements in insulin sensitivity following successful weight loss may revert to pre weight loss levels without continued efforts, even when weight regain remains lower than the initial weight loss^(51–54)^. These findings highlight that weight loss alone may partially account for effects on HbA1c. However, this is not the full explanation, as some almond intervention studies reveal a reduction in HbA1c concentration despite limited body weight loss^(18,43)^. In the present trial, no weight change occurred. This may have limited intervention effects on insulin sensitivity and glycaemia and the potential for almonds to reduce HbA1c concentrations.

Third, the effect of almond consumption on reducing HbA1c concentrations is more consistent among individuals with high baseline values. HbA1c concentrations have not been significantly reduced in healthy individuals administered 1·5 ounces (∼43 g/d) of almonds for ≥ 24 weeks^(19,21)^ even when there was concurrent weight loss^(20)^. Such individuals may have greater capacity to adapt to diets with a diverse nutrient composition, which may explain the lack of significant outcome in this group. Moreover, they are not the primary focus for dietary intervention regimens, as opposed to those exhibiting elevated glycaemic levels. Higher doses of almonds may elicit potent effects on post-prandial glycaemia regardless of baseline HbA1c concentrations^(24)^, but individuals with T2D exhibit increased responsiveness to almond consumption, even at relatively low dosages (e.g. 20 g/d^(10)^). Even individuals with HbA1c concentrations in the prediabetes range exhibit a less pronounced response to the effects of almonds compared with individuals with established T2D. A well-designed trial indicated that individuals with prediabetes (mean HbA1c 5·8 % ± 0·6) who consumed 20 % of their total energy intake from almonds for 16 weeks did not experience significant alterations in HbA1c concentrations^(23)^. In contrast, those diagnosed with T2D (mean HbA1c 7·7 % ± 1·2) who followed a similar dietary regimen, consuming 20 % of their total energy intake from almonds over 24 weeks, exhibited notable changes in HbA1c concentrations^(22)^. In the present trial, the study population included individuals with both prediabetes and T2D, stratified by the cut point HbA1c of 6·4 % (A: 24 prediabetes + 15 type 2 diabetes; C: 30 prediabetes + 12 type 2 diabetes). *Post hoc*, sub-analyses focusing solely on individuals with T2D revealed a non-statistically significant tendency towards lower HbA1c concentrations in the almond group compared with the control group at week 16 (*P* = 0·08). Intriguingly, those subpopulations (T2D) also exhibited a trend of lower body weight in the almond group compared with the control group at week 16 (*P* = 0·09), thereby providing additional evidence supporting the potential synergistic effects of concurrent weight loss with almond consumption on HbA1c concentrations. However, prediabetes/diabetes-specific differences were not considered *a priori* when this study was designed.

Fourth, ethnicity or race may contribute to disparities observed in the effectiveness of almond consumption for reducing HbA1c concentrations. Effects of almond intake on HbA1c concentrations have been more consistent among Asian Indians and Asian Chinese^(18,22,26,42,43)^. South Asians may have lower *β*-cell function which could compromise their ability to compensate for higher circulating glucose concentrations compared with other ethnic groups^(55)^. It is unclear whether this biological variation may be associated with the glycaemic responses to lifestyle interventions more generally^(56–58)^. A systematic review and meta-analysis of randomised controlled trials investigating lifestyle weight-loss interventions revealed differential beneficial effects on HbA1c concentrations across various ethnicities with T2D, notably among Asians as well as Caucasians compared to Black/African or Hispanic groups^(59)^. Although there is no direct evidence on whether ethnicity or race affects responses to almonds differently, it is possible that the absence of an effect on HbA1c in the study by Wein *et al.*, a methodologically strong study, may be explained by dilution due to the diverse ethnic backgrounds of its participants (e.g. Caucasian (38 %), African American (35 %), Hispanic (14 %) and Asian (12 %) participants)^(23)^, while a significant reduction was observed in a similar trial of mainly South Asians^(22)^. The present study assessed predominantly Caucasian individuals (81 %) so may not have used the most sensitive population to test the effects of almonds on HbA1c concentrations.

Despite the fact that nut consumption generally results in higher daily energy intake, epidemiological studies have consistently demonstrated that regular nut consumption is associated with a reduced risk of weight gain over time and a decreased likelihood of developing overweight or obesity as compared to infrequent or no nut consumption^(60,61)^. Indeed, meta-analyses of prospective cohort studies and randomised controlled trials have documented an inverse relationship between the frequency of nut consumption and the risk of weight gain and obesity^(62–64)^, with almonds being particularly notable in this regard^(65)^. These observations have been largely corroborated by clinical trials^(66,67)^. In line with prior findings, individuals enrolled in this study that were assigned to almond intake had higher energy intake (360 kcal/d (95 % CI 156, 564), whereas those in the control group increased intake by only 82 kcal (95 % CI −150, 248). No significant change of body weight was noted in either group, consistent with findings from a systematic review and meta-analyses that indicated the association between nut consumption and weight maintenance is independent of dietary instructions^(68)^. Weight maintenance in the almond group may be attributed to energy compensation from other food sources^(66,67)^, limited bioavailability of energy from almonds^(69,70)^ and/or their potential enhancement of resting energy expenditure^(66)^. Recent systematic reviews and meta-analyses suggest that the first two factors may exert a stronger influence^(71,72)^.

Findings from the 2001–2010 National Health and Nutrition Examination Survey indicate that total diet quality, assessed by the HEI-2010 score, was approximately 15 points higher in almond consumers in comparison with non-consumers^(73)^. A randomised trial has similarly documented a notable enhancement in HEI score with ingestion of almonds^(74)^. In our study, the improvement of diet quality was in accordance with previous trials with a 17 % increment (8·4, 95 % CI 4·1, 12·4) in HEI score. Consumption of low-nutrient dense snacks 2–3 occasions per day is associated with low HEI scores (≤ 59 out of 100), but replacing these typical snacks with almonds significantly increases the HEI score to 70^(75–77)^. Participants enrolled in this study habitually consumed one or more snacks with low nutrient density on a daily basis. This may contribute to their low baseline HEI scores. However, the trial demonstrated that the addition of almonds to their diet, including replacement of one snacking occasion per day, resulted in a significant increase in the HEI score, albeit, only bringing it closer to the USA population average^(34)^.

Assessment of the hedonic ratings of the intervention foods over time is crucial as it relates to the feasibility of chronic inclusion of the foods in the diet. In this study, only a slight reduction in ratings for almonds was noted over time, whereas a more substantial decline was observed for pretzels. This pattern of hedonic shifts aligns with prior research indicating that nuts have low susceptibility to food monotony effects,^(78–80)^ which could facilitate their chronic consumption.

Some limitations from this trial deserve comment. First, there is no quantitative biomarker linked uniquely with almonds to assess compliance with the intervention. This hampers determination of the degree of compliance and the potential effect size of the intervention. Nevertheless, monitoring participants’ circulating *α*-tocopherol concentrations can be useful, even if this micronutrient is not entirely unique to almonds. A randomised controlled feeding trial has shown a dose–response increase in blood *α*-tocopherol concentrations following the consumption of increasing amounts of a single *α*-tocopherol-rich food, such as almonds^(81)^. While the magnitude of the absolute increment varied across individuals, the dose–response relationship observed between plasma *α*-tocopherol concentrations and almond intake offers confidence in using vitamin E as a marker of almond intake compliance. Second, the absence of a controlled feeding period before intervention could have impaired the ability to assess the full impact of the dietary intervention. However, according to the self-reported baseline dietary data, the estimated customary daily servings from the ‘Nuts and Seeds’ food category were significantly lower than the provided almonds during the intervention and did not exhibit variance between groups (0·4 and 0·9 servings in the almond and control groups, respectively, compared with 4·0 servings in the almond group during the intervention). In addition, baseline vitamin E intake was aligned with the average USA intake values where 70 % of the population does not report intake of nuts and seeds. Further, baseline values were lower than the maximum vitamin E intake for a diet without any nuts and seeds according to National Health and Nutrition Examination Survey data^(82)^, which indicates the majority of our participants were not frequent nut consumers. Third, participants were asked to maintain their typical level of physical activity. However, this was not measured and the degree to which a change in this behaviour may have contributed to the stability of body weight in the almond group is unknown. Finally, it should be noted that the *post hoc* sub-analyses conducted were not pre-specified, thus warranting careful interpretation of the findings.

The strengths of the current trial lie in addressing key limitations of prior studies. First, the primary eligibility criteria included elevated HbA1c concentrations, ensuring that the target population consisted of those most likely to respond and benefit from the intervention. Second, the sample size was determined based on a primary outcome, HbA1c, to ensure adequate statistical power to detect changes. Third, the intervention duration was 16 weeks allowing adequate time for measurement of HbA1c concentration changes. Fourth, the selected quantity of almonds was greater than that used in some trials, ensuring an adequate dosage while remaining applicable to the broader population. Lastly, the objective documentation of compliance enhances confidence in the derived conclusions.

### Conclusion

The current findings indicate that incorporating 2 ounces of raw almonds into the customary diet of adults with elevated HbA1c concentrations does not result in a reduction of HbA1c concentration, nor does it improve short-term or longer-term glycaemic or cardiovascular indices. However, future studies should clarify the potentially mitigating influence of background dietary/lifestyle patterns and HbA1c concentrations as well as ethnicity and weight loss on effects of almond consumption on health outcomes. Evidence indicates that their inclusion in the diet is well-tolerated, increases diet quality and does not promote weight gain.

## Supporting information

Huang et al. supplementary material Huang et al. supplementary material

## Supplementary material

For supplementary material/s referred to in this article, please visit https://doi.org/10.1017/S0007114524001053
