# Supplementary material for: Effects of daily almond consumption on glycaemia in adults with elevated risk for diabetes: a randomised controlled trial
Source: Br J Nutr. 2024 Oct 21;132(10):1289–99. doi: 10.1017/S0007114524001053 (PMC11646672; doi:10.1017/S0007114524001053)
Supplement: Huang et al. supplementary material [file S0007114524001053sup001.docx]

**SUPPLEMENTARY MATERIAL**

Appendix Table 1: Post-hoc subgroup analyses on individuals with T2DM and prediabetes based on their baseline HbA1c concentrations. All data are presented as mean (SEM) with each unit listed.

Appendix Table 2: Body weight and BMI remained unchanged in both the almond group and the control group, as measured every 4 weeks. All data are presented as mean (SEM) with each unit listed. To convert body weight from kilogram to pounds, lbs = 2.204*kg.

Appendix Table 3: Body composition measured by dual x-ray absorptiometry (DEXA). All data are presented as mean (SEM) with each unit listed.

Appendix Table 4 : Data measured by continuous glucose monitoring (CGM). All data are presented as mean (SEM) with each unit listed.

Appendix Table 5: Dietary data measured by 24-hr dietary recalls. All data are presented as mean (SEM) with each unit listed.

Appendix Table 6: Plasma α-tocopherol and γ-tocopherol levels measured week 0, 8 and 16. All data are presented as mean (SEM).

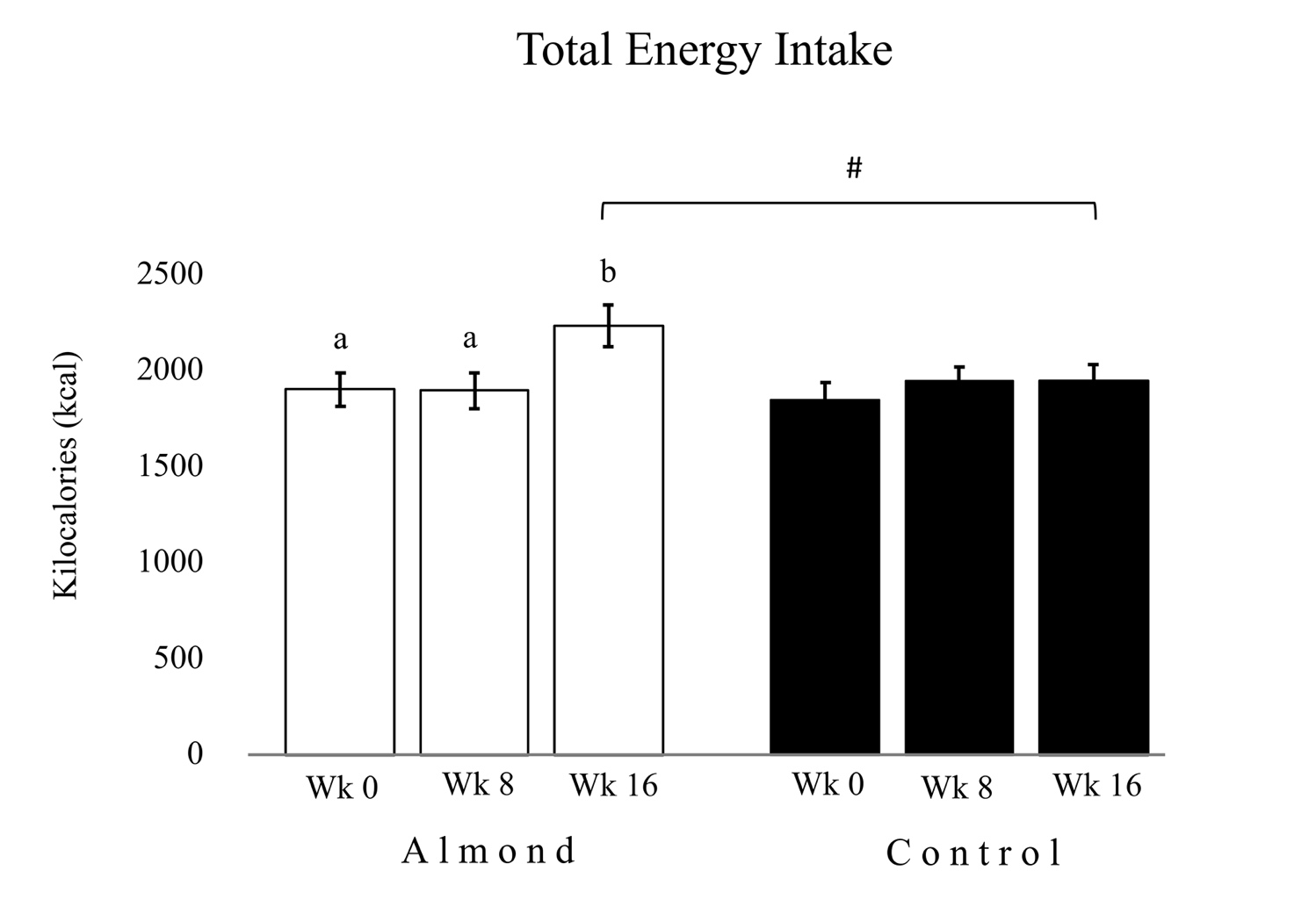


Appendix Figure 1: Total energy intake was significantly higher at week 16 compared to week 0 within the almond group (n=39) and to the control group (n=42). Data for total energy intake are presented as mean ± SEM, with units expressed as kilocalories (kcal). To convert unit from kcal to kJ, kJ=4.18*kcal. □ white box as almond group; ■ black box as control group. ^ab^ Different letters indicate a significant difference compared with week 0 within a group. ^#^ Symbol indicates a significant difference between groups at the same time point. Significance is defined as P<0.05.
